# Demystifying speckle field interference microscopy

**DOI:** 10.1038/s41598-022-14739-0

**Published:** 2022-06-27

**Authors:** Azeem Ahmad, Nikhil Jayakumar, Balpreet Singh Ahluwalia

**Affiliations:** 1 Department of Physics and Technology, UiT The Arctic University of Norway, 9037 Tromsø, Norway; 2 Department of Clinical Science, Intervention and Technology Karolinska Institute, and Center for Fetal Medicine, Karolinska University Hospital, 17177 Stockholm, Sweden

**Keywords:** Applied optics, Lasers, LEDs and light sources, Optical physics, Optical techniques

## Abstract

Dynamic speckle illumination (DSI) has recently attracted strong attention in the field of biomedical imaging as it pushes the limits of interference microscopy (IM) in terms of phase sensitivity, and spatial and temporal resolution compared to conventional light source illumination. To date, despite conspicuous advantages, it has not been extensively implemented in the field of phase imaging due to inadequate understanding of interference fringe formation, which is challenging to obtain in dynamic speckle illumination interference microscopy (DSI-IM). The present article provides the basic understanding of DSI through both simulation and experiments that is essential to build interference microscopy systems such as quantitative phase microscopy, digital holographic microscopy and optical coherence tomography. Using the developed understanding of DSI, we demonstrated its capabilities which enables the use of non-identical objective lenses in both arms of the interferometer and opens the flexibility to use user-defined microscope objective lens for scalable field of view and resolution phase imaging. It is contrary to the present understanding which forces us to use identical objective lenses in conventional IM system and limits the applicability of the system for fixed objective lens. In addition, it is also demonstrated that the interference fringes are not washed out over a large range of optical path difference (OPD) between the object and the reference arm providing competitive edge over low temporal coherence light source based IM system. The theory and explanation developed here would enable wider penetration of DSI-IM for applications in biology and material sciences.

## Introduction

During the last few decades, speckle interferometry has been widely used in the fields of electronic speckle pattern interferometry (ESPI) and speckle shearing interferometry (SSI) to measure the in-plane and out of plane displacement and vibration of the objects with optically rough surfaces^1,2^. Speckle field illumination has also been implemented in various other fields of optical imaging such as structured illumination microscopy^3^, laser speckle contrast imaging (LSCI)^4^, near-field Fourier ptychography^5^, rotating coherent scattering (ROCS) microscopy^6^, polarization holographic microscopy^7^ etc. Recently, temporally varying or dynamic speckle illumination (DSI), which effectively reduces/removes the speckle noise from the images, has been employed in optical profilometry and quantitative phase microscopy (QPM) of biological specimens^8–16^. This type of illumination has high temporal and low spatial coherence properties unlike conventional light sources such as halogen lamp, LEDs and lasers. DSI has several advantages over aforementioned light sources in interference microscopy (IM) such as high spatial phase sensitivity, high resolution and single shot as illustrated in Supplementary Fig. S1^17^. Various IM systems have been proposed in the domain of biomedical imaging applications in the past^18–24^.

Despite its edge over the conventional light sources, implementation of dynamic speckle illumination interference microscopy (DSI-IM) is limited, possibly due to lack of in-depth understanding. In DSI-IM, generation of correlated speckle field is mandatory to form interference pattern when the reference and the object arm speckle fields are superimposed to each other^13^. Therefore, there exist some common misconceptions about the experimental conditions under which the interference fringes in DSI-IM system are obtained^12–14^, such as:

- Use of identical objective lenses in both the object and the reference arms of the interference microscopy system to generate correlated speckle fields,
- Field of view (FOV) fixed and dependent on pre-chosen imaging objective lens,
- Short optical path difference (OPD) adjustment range between the object and the reference arm of the IM system to observe interference pattern at the detector,
- Dependency on diffraction grating in the reference arm to obtain high density fringes over a large field of view (FOV) of the camera.

These doubts also bring the following questions into the discussion:

1. What are the underlying conditions behind the formation of interference fringes in DSI-IM system?
2. Does DSI-IM follow the laws of conventional interferometry in terms of interference fringe’s shape of the resultant pattern?

The lack of in-depth theoretical understanding behind the formation of interference fringes in DSI-IM system restricts the widespread penetration of DSI in the field of phase microscopy. In addition, the underlying experimental conditions to obtain interference fringes in DSI-IM have not been convincingly covered previously. The present article provides the theoretical framework of the formation of interference fringes in DSI-IM resolving previous ambiguities such as use of identical objective lens for the formation of interference fringes and creating of high-density fringes via grating. First, both systematic simulation and experimental studies are performed to understand the conditions required to achieve stable interference patterns in DSI-IM. This is done by superimposing simulated correlated and uncorrelated speckle fields and then studying their effect on the resultant intensity pattern. Similar studies are performed experimentally and are found to be in a good agreement with the simulation findings. It is observed that the speckle fields being overlapped must be correlated and unshifted with respect to each other to obtain high contrast interference fringes in DSI-IM.

It is demonstrated that DSI based IM enables the use of non-identical objective lenses in the object and the reference arm of the system provided the above conditions are satisfied which is contrary to the present understanding. Thus, it provides flexibility to the user to achieve scalable FOV and resolution in the system and demonstrated by performing experiments on USAF chart and placenta tissue samples. Moreover, user defined fringe density (low or high) can easily be obtained in DSI-IM system without using grating in the beam path and provide an ease to implement either single-shot or multi-shot IM. In addition, DSI enables the possibility of extended range of OPD adjustment between the object and the reference arm which is not possible in case of low temporal coherent light source based IM. As live cells require plenty amount of biological medium for keeping themselves alive, which disturbs the OPD significantly and subsequently destroy the interference fringes in IM. Therefore, having this remarkable property, i.e., extended range of OPD adjustment, allows to implement DSI-IM for live cells friendly phase microscopy experiments.

## Simulation studies

### Stable and unstable specklogram

From the statistical theory of optical fields, the speckle fields must be statistically independent or decorrelated to reduce the speckle contrast, i.e., noise, from the images of the test specimens^25^. On the contrary, speckle interferometry requires correlated speckle fields (statistically dependent) which are being superimposed to each other to form stable speckle interferograms or specklograms^12^. The correlation between the speckle fields controls the shape of the resultant intensity patterns. If the speckle fields being superimposed with each other are not correlated (statistically independent), then the resultant intensity pattern represents unstable specklogram.

**Figure 1:**
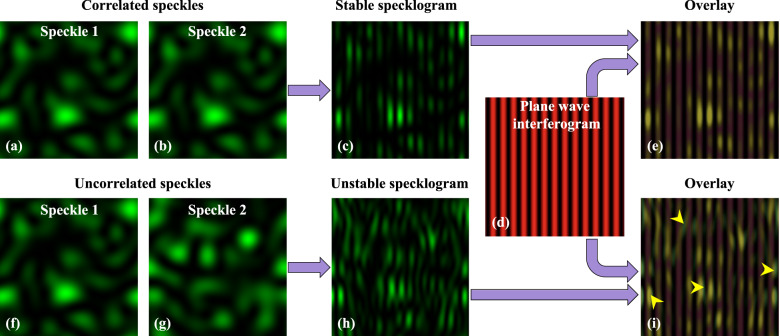
Representation of the superposition of correlated and uncorrelated speckle fields at the detector. (**a**,**b**) Identical speckle fields, (**c**) superposition of identical speckle fields, (**d**) superposition of two plane wavefronts, (**e**) overlay of the correlated speckle fields interferogram and plane wavefronts interferogram. (**f**–**h**) Non-identical speckle fields and their superposition, (**i**) overlay of uncorrelated speckle fields interferogram and plane wavefronts interferogram. The green regions exhibit the speckles in the images. For the correlated case, the interference fringes formed within the speckles follow the orientation of reference fringes and also do not show misalignment in the fringe positions from speckle to speckle. This is represented as a stable specklogram. Whereas, in the uncorrelated case, the fringes present in one speckle are not aligned to the fringes present in other speckles. This leads to the formation of randomly oriented interference fringes in the specklogram. This is termed as an unstable specklogram.

Figure 1 is the pictorial representation of the formation of interference fringes due to the superposition of correlated and uncorrelated speckle patterns. Figure 1a,b exhibit two correlated speckle patterns. The superposition of these speckle patterns does not deform the shape of the resultant intensity pattern and forms nicely oriented specklogram as illustrated in Fig. 1c. It can be seen that the interference fringes do not locally follow the shape and positions of the speckles present in the field of view. On the contrary, the superposition of two uncorrelated speckle fields shown in Fig. 1f,g does not generate nicely oriented interference fringes. It is exhibited in Fig. 1h that the resultant interference fringes have random orientation locally and follow the shape and position of speckles. The deformation in the shape of the resultant interference fringe patterns is exhibited more clearly by overlaying them with the reference interferogram (Fig. 1d) generated due to the superposition of two plane waves. The angle between the plane waves is kept equal to the angle considered during speckle field’s superposition. Figure 1e,i illustrate the overlayed images of the stable and unstable specklograms with the reference interferogram. The fringes in the stable specklogram exactly overlap with the reference fringes. Whereas, for unstable specklogram, the fringes do not overlap with the reference fringes. Some of such locations are exhibited by yellow arrows in Fig. 1i.

### The superposition of correlated and uncorrelated speckle patterns

**Figure 2:**
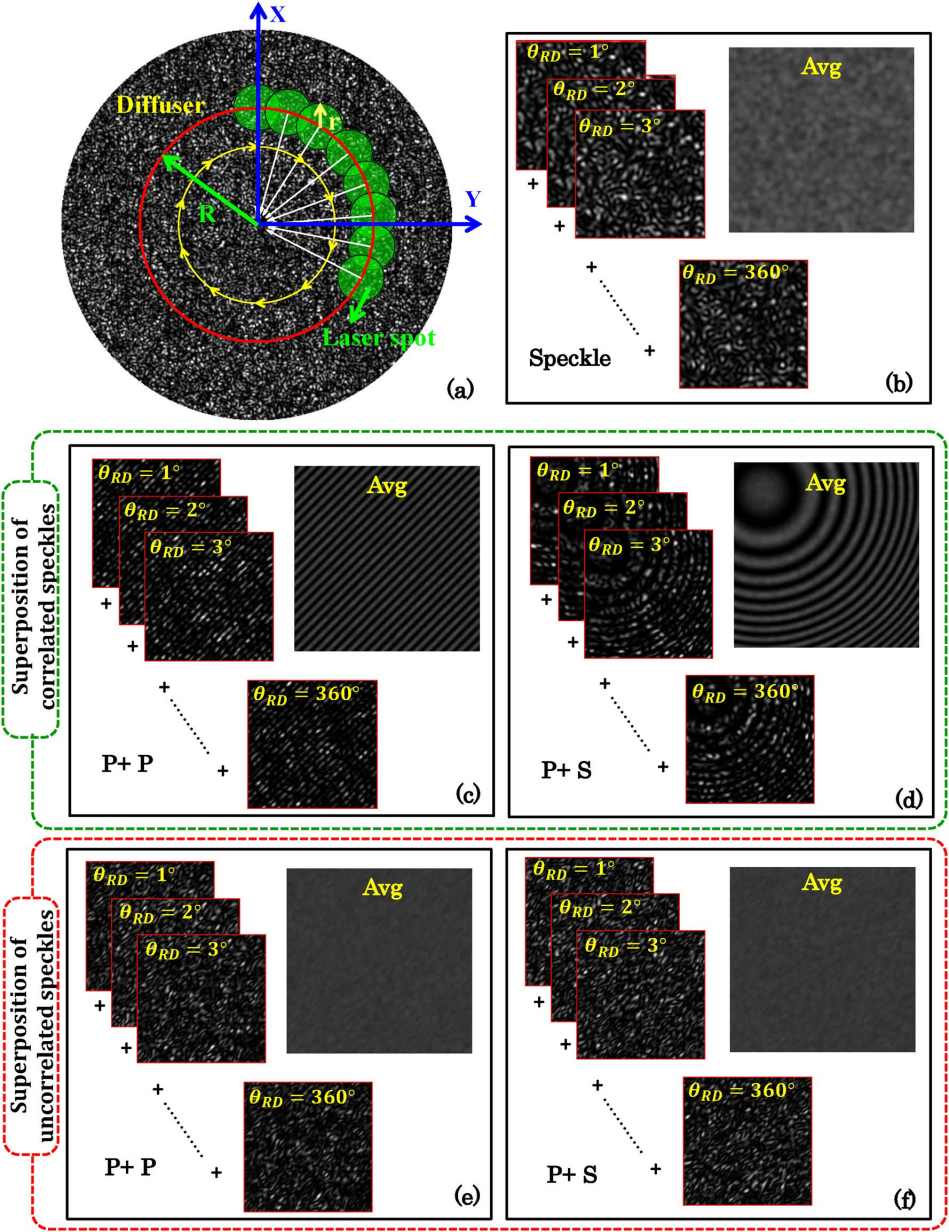
Superposition of correlated and uncorrelated speckle fields. (**a**) Mimicking of the rotating diffuser in the simulation. Green color filled circles represent the laser spot as a function of different rotation angles θ_RD_. Red color solid circle is the circular path (radius R) of the laser spot (radius r) at the diffuser. (**b**) Represents the series of statistically independent speckle patterns corresponding to different rotation angles *θ*_*RD*_ from 0° to 360° in a step of 1° and their average image. (**c**,**d**) illustrate the specklograms and averaged interferograms generated due to the superposition of correlated object and reference speckle fields for plane and spherical global phase fronts, respectively. (**e**,**f**) exhibit the resultant intensity patterns generated due to the superposition of uncorrelated object and reference speckle fields for plane and spherical global phase fronts, respectively. P and S are defined as; P: Plane phase fronts and S: Spherical phase front.

A systematic simulation study is done to understand the effect of the superposition of two correlated or two uncorrelated speckle fields on the resultant intensity patterns. The speckle theory and mathematics of speckle interferometry can be found in Sects. 2 and 3 of the Supplementary Information. First, a rough surface of size of 512 × 512 pixels is generated to mimic the static diffuser and then multiplied by a circular binary mask with opening of diameter D (see Fig. S3), which controls the laser light beam diameter in the simulation. Here, the window size ‘D’ is considered equal to 30 pixels. The details can be found in Sect. 4 of Supplementary Information. The position of the opening in the mask (partially filled green circle) is kept at a distance R from the center of the diffuser as shown in Fig. 2a. The opening in the mask is then rotated along the circular path depicted in red color. The angular position of the mask’s opening is varied from 0° to 360° in a step of 1°. This generates statistically independent speckle fields (S_1_, S_2_, …, S_n_) corresponding to different rotation angles of the opening. Figure 2b illustrates the statistically independent speckle patterns (contrast = 0.98) corresponding to the rotation angles ‘*θ*_*RD*_’ from 0° to 360° in a step of 1°. The speckle noise can be reduced by averaging such large number of statistically independent speckle fields. The average of these statistically independent speckle patterns is shown in Fig. 2b which is having very low contrast equal to 0.13.

Since it is well known from Fourier transform’s shifting property that the non-centric position of the opening affects the resultant speckle field by introducing an extra phase factor in the calculation^26^. Therefore, here before taking the Fourier transform of the simulated rough surface to generate speckle pattern, the region of the diffuser falling in the opening region of the mask is shifted to the center of the diffuser. This is done to mimic the experimental condition in which the light beam is aligned to the optical components of the phase microscope and diffuser is rotating.

Next, for dynamic speckle illumination interferometry, two correlated or uncorrelated speckle fields are generated by simulating two identical or non-identical diffusers, respectively. The correlated or uncorrelated speckle fields are then superimposed at an angle ‘θ’ to study their effect on the resultant intensity pattern also called specklogram. More details are provided in Sect. 5 of the Supplementary Information. This situation is analogous to the optical interference of two identical or random speckle fields.

Figure 2c,e illustrate the specklograms corresponding to the rotation angles ‘θ_RD_’ from 0° to 360° in a step of 1° for the correlated and uncorrelated speckle fields, respectively. The global phase fronts are considered plane for both the speckle fields (R and O) being superimposed to generate specklogram. The average images of 360 specklograms corresponding to correlated and uncorrelated speckle fields are also shown in Fig. 2c,e, respectively. The average of the specklograms formed due to correlated speckle fields, generates clean interferogram. Next, two speckle fields having global plane and spherical wavefronts are superimposed to generate corresponding specklogram. The details are given in Sect. 5 of the Supplementary Information. In the case of correlated speckle fields, the average of 360 specklogram generates clean and nicely oriented off-centered circular fringes as shown in Fig. 2d. For the uncorrelated case, fringe pattern is not formed and average image with approximately uniform intensity is generated as illustrated in Fig. 2e,f. In the uncorrelated case, two statistically independent speckle fields are generated using *rand* MATLAB command. This situation is analogous to the consideration of axial diffraction in one of the speckle fields which changes the speckle pattern and generates statistically independent speckle field outside the longitudinal coherence length of instantaneous speckles. Thus, to generate high contrast and clean interference fringes in speckle interferometry setups, the fields must be correlated to each other. This concludes that the shape of the global phase fronts does not reduce the quality of the specklogram as long as the speckle patterns are correlated. In addition, interference pattern formation does not depend on the shape of the global phase fronts. It only changes the fringe shape of the resultant interference pattern. Thus, speckle interferometry follows the laws of conventional interferometry in terms of fringe shapes provided the fields are correlated.

### Superposition of the shifted speckle fields

In this section, the effect of the superposition of a speckle field with its shifted version on the resultant intensity is systematically studied. This situation is analogous to the experimental condition where both the speckle fields (object and reference) originated from the same source do not exactly overlap to each other due to slight misalignment in the optical setup. The details about the experimental conditions are discussed in section “Experimental results”. This study is conducted with the speckle fields covering an area of 512 × 512 pixels with two different speckle sizes of 15 pixels and 30 pixels. To generate two different speckle sizes of 15 pixels and 30 pixels, the window size ‘D’ (see Fig. S3) is considered equal to 30 pixels and 15 pixels, respectively.

**Figure 3:**
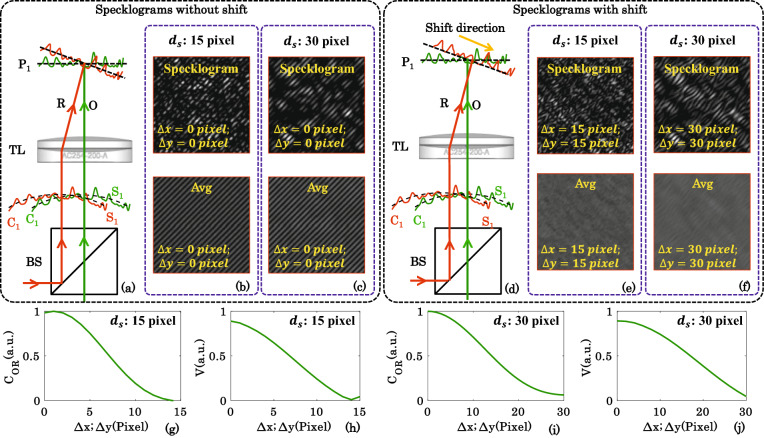
Correlation study with shift only in one speckle fields and speckle size. The correlation between the identical speckle fields as a function of shift of one of the speckle fields being superimposed to generate resultant intensity pattern. (**a**–**c**) Represent the optical configuration, specklogram and averaged interferogram corresponding to the superposition of unshifted correlated speckle fields of speckle sizes 15 pixels and 30 pixels, respectively. (**d**–**f**) Represent the optical configuration, specklogram and averaged interferogram corresponding to the superposition of shifted correlated speckle fields of speckle sizes of 15 pixels and 30 pixels, respectively. (**g**,**i**) Illustrate the variation of correlation values between the speckle fields as a function of the shift in a step of 1 pixel corresponding to speckle fields of speckle sizes of 15 pixels and 30 pixels corresponding to the window sizes ‘D’ of 30 pixels and 15 pixels, respectively. (**h**,**j**) Corresponding fringe visibility plot obtained from the averaged images/interferograms.

First, a speckle field is superimposed with its shifted version to generate specklogram for both speckle sizes of 15 pixels and 30 pixels. The shifted version is obtained from the copy of the same field. This is done by translating the speckle field along x and y direction in a step of say ∆*x* and ∆*y*. The value of ∆*x* and ∆*y* is varied from zero to the average size of the speckle. Figure 3a–c illustrate the case of superposition of identical unshifted speckle fields at plane P_1_. The ray diagram of the overlap of identical speckle fields (R and O) at plane P1 is shown in Fig. 3a. The specklograms generated due to the superposition of the identical speckle fields of speckle sizes of 15 pixel and 30 pixel are depicted in the upper row of Fig. 3b,c, respectively. The specklograms exhibited in Fig. 3b,c correspond to the rotation angle ‘*θ*_*RD*_’ equal to zero. The rotation angles ‘*θ*_*RD*_’ is defined in the previous section and varied from 0° to 360° in a step of 1° to generate statistically independent specklogram. The average images are then generated by taking the average of 360 specklograms corresponding to both speckle sizes of 15 pixel and 30 pixel and illustrated in Fig. 3b,c. It can be visualized from the average images that the resultant modulated intensity pattern is not affected by the size of the speckle in the case of unshifted speckle fields.

One of the speckle fields is then shifted by 15 pixels and 30 pixels corresponding to the speckle sizes of 15 pixels and 30 pixels, respectively. The ray diagram of the overlap of identical speckle fields (R and O) with a shift at plane P1 is shown in Fig. 3d. These shifted speckle fields are then superimposed with the original/unshifted copy of the speckle field to generate specklograms as shown in Fig. 3e,f. It can be seen from the specklograms shown in Fig. 3e,f that the local fringe orientations follow the shape and position of the speckles, i.e., the fringes are not nicely oriented along a particular direction as explained in the previous section. For the fringe width greater than the size of the speckle (15 pixels), the local fringes are not visible in the resultant intensity pattern. In the case of speckle size of 30 pixels, the local fringes are observed due to the smaller fringe width than the speckle size as depicted in Fig. 3f. The corresponding average images, obtained after averaging 360 specklograms, are illustrated in Fig. 3e,f. Thus, in order to generate interference fringes the fields being superimposed should not be shifted greater than the average size of speckles. In the simulation, high to moderate contrast (i.e., 0.9 to 0.5) interference fringes are only observed until the shift in one of the speckle field is less than the half of the speckle size.

To understand this, one of the speckle fields is shifted sequentially from 0 to the average speckle size in a step of 1 pixel and corresponding specklograms and average images are generated. The values of the correlation between the shifted speckle patterns and the original speckle pattern are then calculated using Supplementary Equation (S3) and plotted as a function of shift. The corresponding average images for different shifts are exhibited in Supplementary Figs. S5 and S6. Figure 3g,i illustrate the correlation maps as a function of shift corresponding to the speckle sizes of 15 pixels and 30 pixels. It can be seen from the correlation map of speckle size of 15 pixels (Fig. 3g), the value of the correlation drops down to approximately zero for the shift equal to the speckle size. On the contrary, the correlation never drops to zero in the case of speckle size of 30 pixels as shown in Fig. 3i. Thus, speckle fields having larger speckle size always have some correlation under shift.

To further confirm this, the visibilities of the average images corresponding to every shift are then calculated and plotted as depicted in Fig. 3h,j. The details to calculate the visibility of an interferogram can be found in Ref.^27^. The visibility curves also follow the similar trend as the correlation plot. However, the visibility of the interferograms does not start from 1 even for the perfectly correlated case. The slight deviation could be due to the insufficient averaging of statistically independent speckle fields being used to generate specklograms. It is also observed that average images (interferograms) have slight local variations in the fringe modulation and can be clearly seen in the Supplementary Fig. S7.

## Experimental results

### Superposition of correlated and uncorrelated speckle fields

First, an experimental study of the simulation work presented in sections “The superposition of correlated and uncorrelated speckle patterns” and “Superposition of the shifted speckle fields” is done. To perform this study, the superposition of correlated and uncorrelated speckle fields at the detector is done experimentally to understand their effect on the resultant intensity pattern. In the object and reference arms 60 ×/0.7NA and 10 ×/0.25NA objective lenses are used to generate object and reference speckle fields, respectively. A piece of silicon wafer is used in both the object and the reference arms of IM to match the reflected intensity of both the beams. In order to generate correlated object and reference speckle fields experimentally, following condition must be satisfied: (1) Si-wafer in the object and the reference arm is within the depth of field of the objective lenses, and (2) precise alignment of both the object and the reference speckle fields within the correlation length.

**Figure 4:**
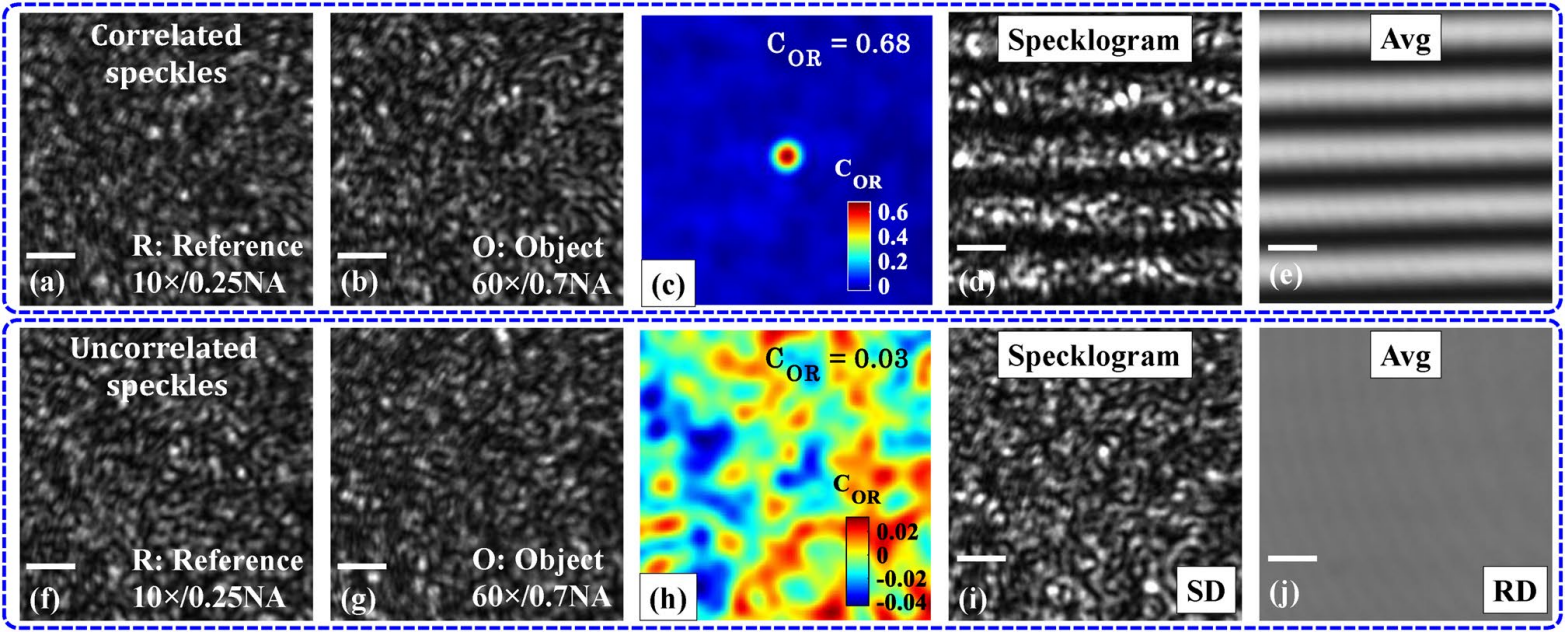
Superposition of the correlated and uncorrelated speckle fields. The objective lenses being employed in the object and the reference arm are 60 ×/0.7NA and 10 ×/0.25NA, respectively. (**a**,**b**) Correlated object and reference speckle fields. (**c**) Normalized correlation map. In both correlation maps, only 128 × 128 central pixels are considered to clearly demonstrate the correlation peak. (**d**,**e**) Corresponding specklogram (for *SD* stationary diffuser) and interferogram (for *RD* rotating diffuser). (**f**,**g**) Uncorrelated object and reference speckle fields. The decorrelation between the fields is done by translating and tilting the reference arm Si-wafer. (**h**) Corresponding normalized correlation map. (**i**,**j**) Corresponding specklogram (for *SD* stationary diffuser) and fringe free uniform intensity (for *RD* rotating diffuser). The scale bars are represented in solid white color horizontal lines. Scale bar: 50 pixels.

Figure 4a,b represent the correlated object and reference speckle fields recorded by the camera by sequentially blocking the reference and the object arm of the interferometer. The diffuser is kept stationary to generate static speckle patterns. The normalized correlation between both the patterns is then calculated and found to be equal to 0.68. The corresponding normalized correlation map is shown in Fig. 4c. Next, both the object and the reference arms of the IM are unblocked to record the specklogram as illustrated in Fig. 4d. The diffuser is then rotated to remove the speckle noise from the specklogram as explained in the section “The superposition of correlated and uncorrelated speckle patterns”. The rotating diffuser didn’t affect the interference fringes and generated nicely oriented speckle noise free interferograms as depicted in Fig. 4e.

Further, the reference Si-wafer is translated and tilted to introduce decorrelation between the object and reference speckle fields. The decorrelated object and reference speckle fields are illustrated in Fig. 4f,g, respectively. The normalized correlation map is depicted in Fig. 4h and its value is found to be equal to 0.03. It can be seen that the correlation between the speckle fields dropped down by a significant amount compared to the correlated ones. The corresponding specklogram is illustrated in Fig. 4i. It can be visualized that the specklogram generated due to the superposition of uncorrelated speckle fields does not contain fringe like pattern. The diffuser is then rotated to generate temporally varying speckle fields which leads to the formation of uniform intensity (i.e., without interference fringes) over the camera FOV as shown in Fig. 4j. These experimental findings are found to be in a good agreement with the simulation results presented in sections “The superposition of correlated and uncorrelated speckle patterns” and “Superposition of the shifted speckle fields”.

### Correlation study for different microscope objective lenses

Next, four different objective lenses 10 ×/0.25NA, 20 ×/0.45NA, 60 ×/0.7NA and 60 ×/1.2NA are employed in the object arm sequentially to understand their effect on the resultant intensity pattern at the camera. The reference arm objective lens is not changed and always kept 10 ×/0.25NA during all the experimentation. Again, a piece of Si-wafer is used as a sample under the IM system. First, the diffuser is kept stationary to generate object and reference speckle fields by employing the experimental scheme exhibited in Fig. 8. As it is mentioned in the experimental section, the reference mirror/Si-wafer is attached to a three-axis precision control stage: one translation and two angular motions. The reference mirror is adjusted in a way such that both the arms: object and reference, generate correlated speckle fields at the detector. The translation of the reference mirror helps to obtain stable specklograms. The angular motions of mirror controlled the fringe density in the camera FOV as explained in the experimental section.

**Figure 5:**
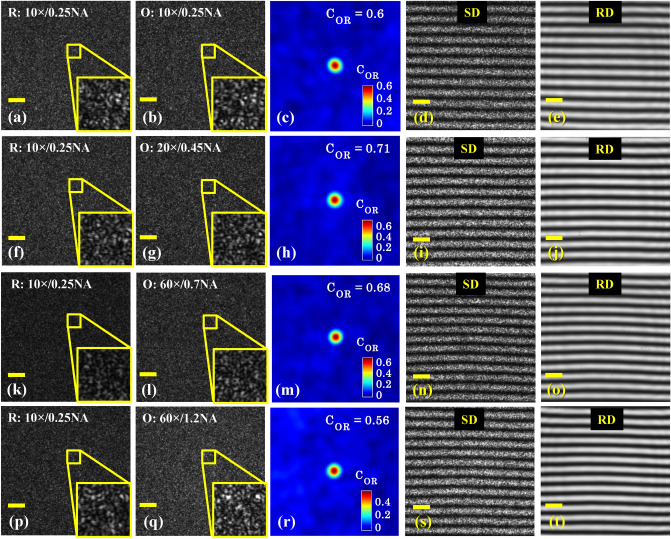
Correlation between the object and reference speckle fields as a function of magnification and numerical aperture. Four different objective lenses 10 ×/0.25NA, 20 ×/0.45NA, 60 ×/0.7NA and 60 ×/1.2NA are sequentially inserted in the object arm keeping fixed reference arm objective lens 10 ×/0.25NA. (**a**,**f**,**k**,**p**) and (**b**,**g**,**l**,**q**) The reference and the object arm speckle fields corresponding to fixed reference objective lens and all four objective lenses in the object arm, respectively. (**c**,**h**,**m**,**r**) illustrate the 2D normalized correlation map between both the speckle fields. In all correlation maps, only 128 × 128 central pixels are considered to clearly demonstrate the correlation peak. (**d**,**i**,**n**,**s**) Superpositions of the object and the reference arm speckle fields also called specklogram corresponding to all four objective lenses in the object arm. The diffuser is kept stationary. (**e**,**j**,**o**,**t**) The temporally averaged patterns of a large numbers of specklograms within the exposure time of the camera. This is achieved by rotating the diffuser at an adequate speed. The scale bars are represented in solid yellow color horizontal lines. Scale bar: 300 pixels.

Figure 5 exhibits the speckle fields in the object and the reference arm of the interferometer and their superposition at the camera for all four objective lenses 10 ×/0.25NA, 20 ×/0.45NA, 60 ×/0.7NA and 60 ×/1.2NA. Figure 5a,f,k,p and b,g,l,q illustrate the speckle fields corresponding to the reference and the object arm for 10 ×/0.25NA, 20 ×/0.45NA, 60 ×/0.7NA and 60 ×/1.2NA objective lenses and represented as R and O. Here, R and O are stand for reference and object arm of the interferometer. The object and the reference arm of the interferometer are sequentially opened in order to record the related speckle fields. The correlation between both the speckle fields for all four objective lenses are calculated using a MATLAB code and listed in Table 1. Figure 5c,h,m,r exhibit their 2D normalized correlation maps, respectively. Further, both the object and the reference arm of the interferometer are opened to generate the specklogram corresponding to all objective lenses as illustrated in Fig. 5d,i,n,s.

**Table 1:** Speckle sizes as a function of objective lens magnification and NA.

S. no.	Objective lens		Average speckle size (pixels)	C_OR_ (a.u.)
	Magnification	Numerical aperture (NA)		
1.	10 ×	0.25	8.8	0.60
2.	20 ×	0.45	8.9	0.71
3.	60 ×	0.70	8.7	0.68
4.	60 ×	1.20	8.2	0.56

It can be clearly visualized that the correlations between the object and the reference arm for all four objective lenses are quite high and leads to the formation of high contrast interference fringes. In order to understand the theory behind obtaining high correlation between both the speckle fields for all four objective lenses in the object arm with a fixed objective lens in the reference arm, first, the average speckle sizes of the speckle fields coming from both the arms are measured. Table 1 exhibits the average speckle sizes of the patterns shown in Fig. 5b,g,l,q for all object arm objective lenses. It can be seen that the average speckle size is measured to be approximately equal for all lenses. This became possible due to the reflection geometry of the present optical configuration, where the speckle field originated from output port of MMF (Fig. 8) is passed twice through both the object and the reference arm objective lenses. The objective lenses, first, demagnified the input speckle field and formed diffraction limited pattern at the sample and the reference mirror. Due to the reflection geometry, the demagnified speckle fields in both the arms are collected by the same objective lens and magnified by the similar amount and speckle size remain unchanged. Thus, the correlation between the speckle fields do not destroy while passing through different objective lenses in the object arm and fixed objective lens in the reference arm and enable the use of non-identical objective lenses in both the arms of the interference setup.

In the transmission geometry, the speckle field is passed only once through the fixed objective lens, therefore, the situation is not identical to the reflection geometry. The switching of the objective lens in the object arm keeping fixed objective lens in the reference arm changes the speckle size of the object arm speckle field when exiting the objective lens and destroy the correlation (i.e., mandatory condition to obtain static/stable specklogram) between both the beams and subsequently the interference fringes. In transmission geometry, identical objective lenses must be used in both the arms in order to satisfy the condition of stable specklogram^14^. Moreover, the distance between the objective lenses and the sensor is also crucial and must be equal to maintain the high correlation between the object and the reference arm speckle fields. If the aforementioned conditions fail to satisfy, it leads to a significant drop in the correlation between the superimposed speckle fields and consequently the fringe contrast of the resultant pattern. Thus, this imposes lots of restrictions for obtaining correlated speckle fields and limits the implementation of IM system only for a single objective lens.

The specklograms illustrated in Fig. 5d,i,n,s are filled with huge speckle noise. The stationary diffuser is then rotated to generate large number of statistically independent specklograms within the exposure time of the camera. The camera exposure time is set to 30 fps. The average images of large number of specklograms corresponding to all four objective lenses 10 ×/0.25NA, 20 ×/0.45NA, 60 ×/0.7NA and 60 ×/1.2NA are presented in Fig. 5e,j,o,t, respectively. It can be seen that the average images called interferogram are free from the speckle noise and coherent noise. Note that the temporally varying speckle field arises due to rotating diffuser does not affect the shape and quality of the resultant intensity patterns at the detector for all four objective lenses. This is found to be in a good agreement with the simulated results.

### Extended range of OPD adjustment

**Figure 6:**
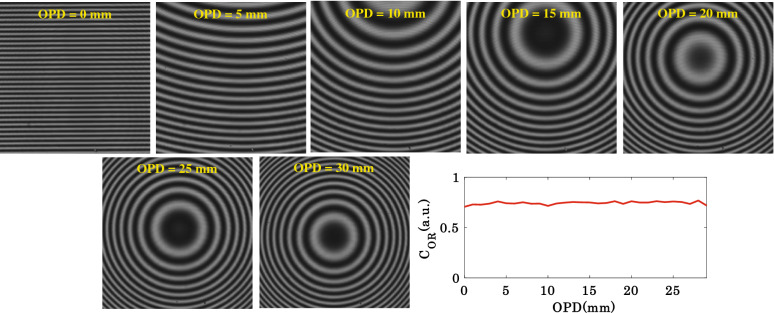
The interferograms generated due to the superposition of correlated speckle fields in both the object and the reference arms of the interferometer as a function of the optical path difference (OPD).

In order to perform this study, identical 10 ×/0.25NA objective lenses are used in both the object and the reference arms of the system. As DSI has high temporal and low spatial coherence length. Therefore, to understand the advantage of high temporal coherence length of DSI, the reference arm unit (objective lens MO_2_ and mirror M together) is translated to introduce OPD between the object and the reference arm. The OPD is increased from 0 to 35 mm in a step of 1 mm. It can be visualized that non-zero OPD does not washout the interference pattern due to the generation of correlated speckle fields in the object and the reference arms. This is contrary to the low temporal coherence light source based IM systems. The non-zero OPD only generates curved or circular fringes at the camera as illustrated in Fig. 6. The line profile illustrates the variation of the normalized correlation between the object and the reference speckle fields as a function OPD. It can be seen that the correlation does not drop between the speckle fields as a function of increased OPD. This is due to the high temporal coherence length and double pass of the speckle field through the same objective lens in the reference arm, which keeps the speckle fields correlated with the object arm speckle field. The double pass through the objective lens cancels the effect of non-zero OPD on the speckle field and keeps the speckle size and shape unchanged in the reference arm. The correlation values between the object and the reference speckle fields as a function of OPD are given in the Supplementary Table S1.

### Phase imaging with scalable FOV and resolution

In the previous section, it has been successfully demonstrated that different magnification and numerical aperture objective lenses can used in the object arm of the IM system without affecting the quality of the resultant interference pattern. The fringe density of the interference pattern is kept less to clearly show the potential of the DSI in IM system. However, this is not only limited to low fringe density of the interference pattern. The fringe density can be made very high to implement single shot IM. The fringe density can be increased by tilting the reference mirror. The tilting of the mirror decorrelates the object and reference speckle fields, which can be adjusted by translating the mirror either towards or away from MO_1_. The high fringe density interferogram of the USAF chart and its single shot recovered phase map is illustrated in Supplementary Fig. S8.

To demonstrate the capability of DSI in terms of enabling scalable FOV and resolution possibility in IM, experiments are conducted on USAF resolution test target (Thorlabs: part # R3L3S1N) and placenta tissue sample. The placenta tissue sample is sectioned into 1 μm thick cryosection (EMUC6 ultramicrotome, Leica Microsystems, Vienna, Austria), and placed on top of a reflecting Si substrate. The sample is placed inside a polydimethylsiloxane (PDMS) chamber of thickness of 500 μm and opening size of 5 mm × 5 mm as illustrated in the experimental setup (Fig. 8). Thereafter, 50 μl volume of phosphate-buffered saline (PBS) is put inside the PDMS chamber to keep the tissue sample hydrated and sealed with a cover glass of # 1.5 to enable the use of objective lenses for imaging. The details of the tissue sample preparation can be found in Ref.^17^. The Regional Committee for Medical and Health Research Ethics of Norway (REK_nord) approved the ethical clearance and informed consent was obtained from all participants for conducting the experiments on the tissue samples and all methods were performed in accordance with the relevant guidelines and regulations.

**Figure 7:**
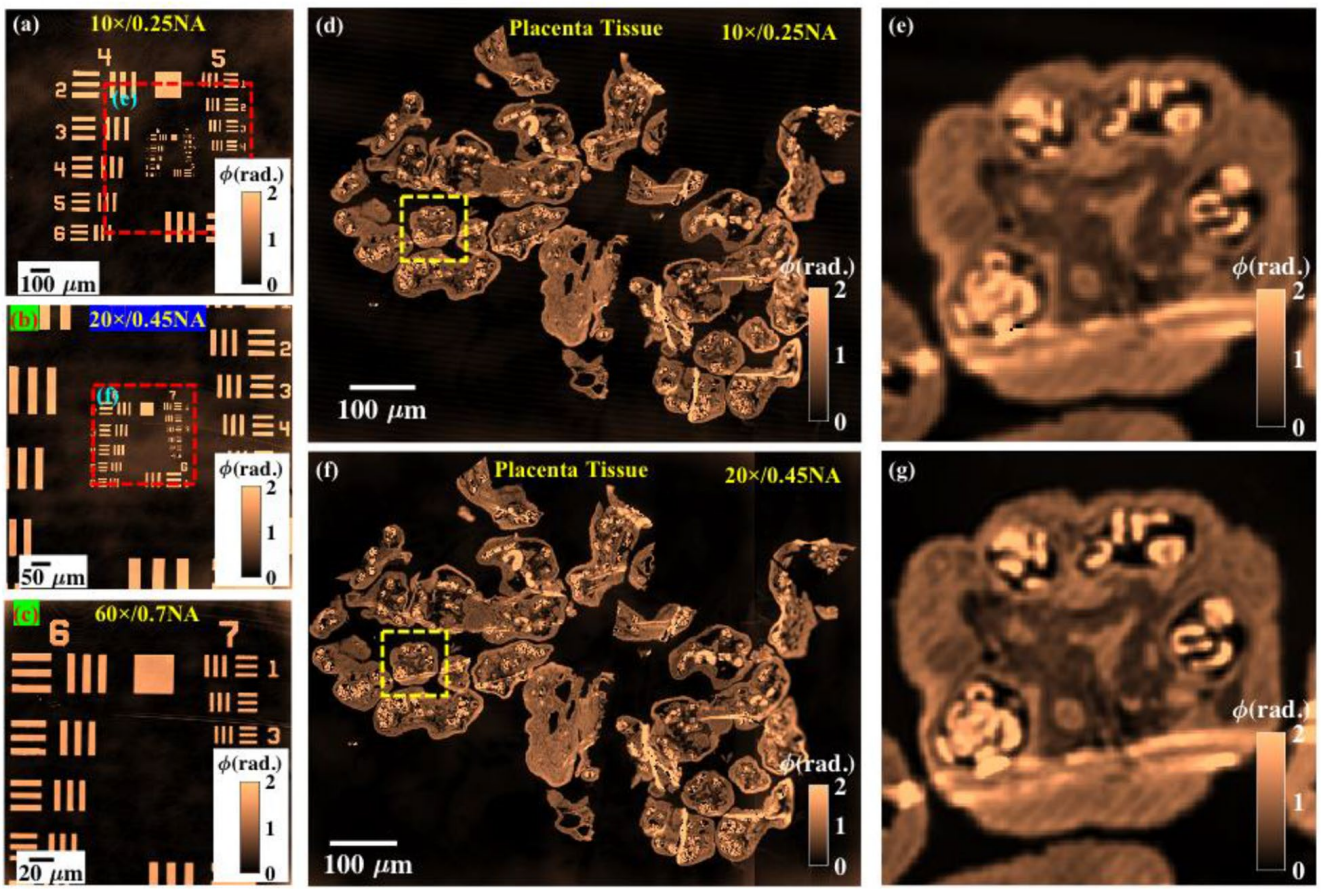
Scalable FOV and resolution phase imaging. DSI generates scalable FOV and resolution quantitative phase images without changing the reference objective lens. (**a**–**c**) Recovered phase maps of USAF resolution chart while using 10 ×/0.25NA, 20 ×/0.45NA and 60 ×/0.7NA objective lens in the object arm, respectively. (**d**–**g**) Reconstructed phase images and their zoomed views of placenta tissues samples corresponding to 10 ×/0.25NA and 20 ×/0.45NA objective lenses, respectively. The scale bars are in micrometer.

For USAF chart, experiments are performed using 10 ×/0.25NA, 20 ×/0.45NA and 60 ×/0.7NA objective lenses in the object arm keeping 10 ×/0.25NA objective lens in the reference arm. Similarly, for placenta tissues samples, interferometric data is recorded with only two objective lenses 10 ×/0.25NA and 20 ×/0.45NA to exhibit large FOV imaging. For both the sample, random phase shifted interferograms are recorded and then PCA based reconstruction algorithm is implemented for their phase recovery^28^. The phase shift between the interferograms is introduced by shifting the reference mirror using piezo transducer with nanometer precision. Figure 7a–c illustrate the recovered phase maps of the resolution chart corresponding to 10 ×/0.25NA, 20 ×/0.45NA and 60 ×/0.7NA objective lenses, respectively. It can be seen that the recovered phase maps suffer from slowly varying background phase due to the presence of minute optical aberrations in the IM system. This can be corrected numerically by employing any phase aberration compensation algorithm. The red dotted boxes in Fig. 7a,b illustrate the FOVs of 20 ×/0.45NA and 60 ×/0.7NA objective lenses, respectively.

The recovered phase images of placenta tissue samples corresponding to 10 ×/0.25NA and 20 ×/0.45NA objective lenses are exhibited in Fig. 7d,f, respectively. The zoomed views of the regions marked with yellow dotted boxes are illustrated in Fig. 7e,g. It can be clearly visualized that 20 ×/0.45NA generates higher resolution phase image of the sample compared to 10 ×/0.25NA. It can be visualized that the recovered phase maps do not suffer from the speckle noise and coherent noise. The stripe patterns observed in Fig. 7e,g are caused by the microtome blades during sample preparation. The high spatial phase sensitive phase images obtained by DSI also enables the visualization of sectioning artifacts on the specimens over the whole camera FOV. In addition, it is demonstrated that DSI provides the possibility of obtaining scalable FOV and resolution in IM system.

## Discussion and conclusion

In the present work, systematic simulation and experimental studies are done to understand physics behind achieving the interference in DSI-IM system. It is observed that DSI forms stable interference signal under rotation of the diffuser if the speckle fields being overlapped are correlated with each other. On the contrary, the superposition of uncorrelated speckle fields does not form nicely oriented fringes in the resultant specklogram and washes out the interference pattern when the diffuser is rotated.

Further, the superposition of a speckle field and its identical spatially shifted version is done to understand its effect on the resultant intensity pattern. It is found that stable interference fringes are formed only if the shift between the speckle fields being superimposed to each other is smaller than the average speckle size. It is observed that high to moderate contrast (i.e., 0.9 to 0.5) interference fringes are formed for the shift less than or equal to half of the speckle size. Interestingly, speckle fields having bigger speckle sizes always exhibited little correlation and lead to non-zero correlation between them.

DSI has high temporal coherence (TC) length almost equal to the TC length of the parent laser light source and low spatial coherence (SC) length depending on the source size. High TC length of DSI helps to achieve the interference pattern quickly in IM systems compared to low TC light sources such as halogen lamp, light emitting diodes etc. DSI can also be used to balance the object and the reference arm of reflection IM systems, to obtain interference patterns with low TC light sources. In addition, DSI derived from a high TC length light source enables the user defined magnification and resolution in the IM system. Moreover, DSI provides high spatial phase sensitivity comparable to low TC length light source in IM system^17^. Most of the biological applications require a highly spatial phase sensitive IM phase microscope with the possibility of changing objective lenses of different magnifications and numerical apertures in the object arm. This would make DSI-IM system a suitable candidate for several biological applications.

Here, it is important to highlight that changing the objective lens only in the object arm disturbs the zero OPD between the object and the reference beam. As the OPD between both the beams must be smaller than the TC length of light source to observe interference pattern at the detector. Therefore, low TC light sources cannot form interference fringes in such unbalanced interferometric system. On the contrary, DSI being high TC in nature can form the interference fringes even in an unbalanced IM configuration provided both the fields reaching at the detector are correlated and unshifted. Moreover, the photon degeneracy of DSI is very high compared to halogen lamp or LEDs^29,30^. The high photon degeneracy can also be obtained by employing direct narrow band laser at the cost of unwanted interference fringes due to the coherent superposition of multiple reflections coming from surfaces of the optical components^14,17,31^. As a consequence, it reduces the spatial phase sensitivity and measurement accuracy of the system.

Thanks to the reflection geometry of IM employed with DSI, which provides the freedom of moderate translation (several mm) of the reference arm (mirror and objective lens both) without affecting the contrast of the interference fringes (Fig. 6). Thus, the precise positioning of the object and the reference arm within few tens of micrometers is not required as in the case of low TC light source. DSI-IM generates straight, circular and curved fringes depending on the position of the reference arm (Fig. 6). In addition, the fringe density can be easily tuned from extremely low to extremely high over the whole camera FOV without affecting the interference fringe quality (see Supplementary Fig. S8). Thus, DSI enables both single-shot and multi-shot quantitative phase recovery of the specimens under test. In order to support our claim, the scalable FOV with scalable resolution quantitative phase imaging of a standard USAF resolution test target (in both single-shot and multi-shot) and placenta tissue samples (in multi-shot) is demonstrated. We believe the present work would provide a deeper understanding of DSI-IM for the development of such systems and their potential use for future applications.

## Materials and methods

### Experimental setup

**Figure 8:**
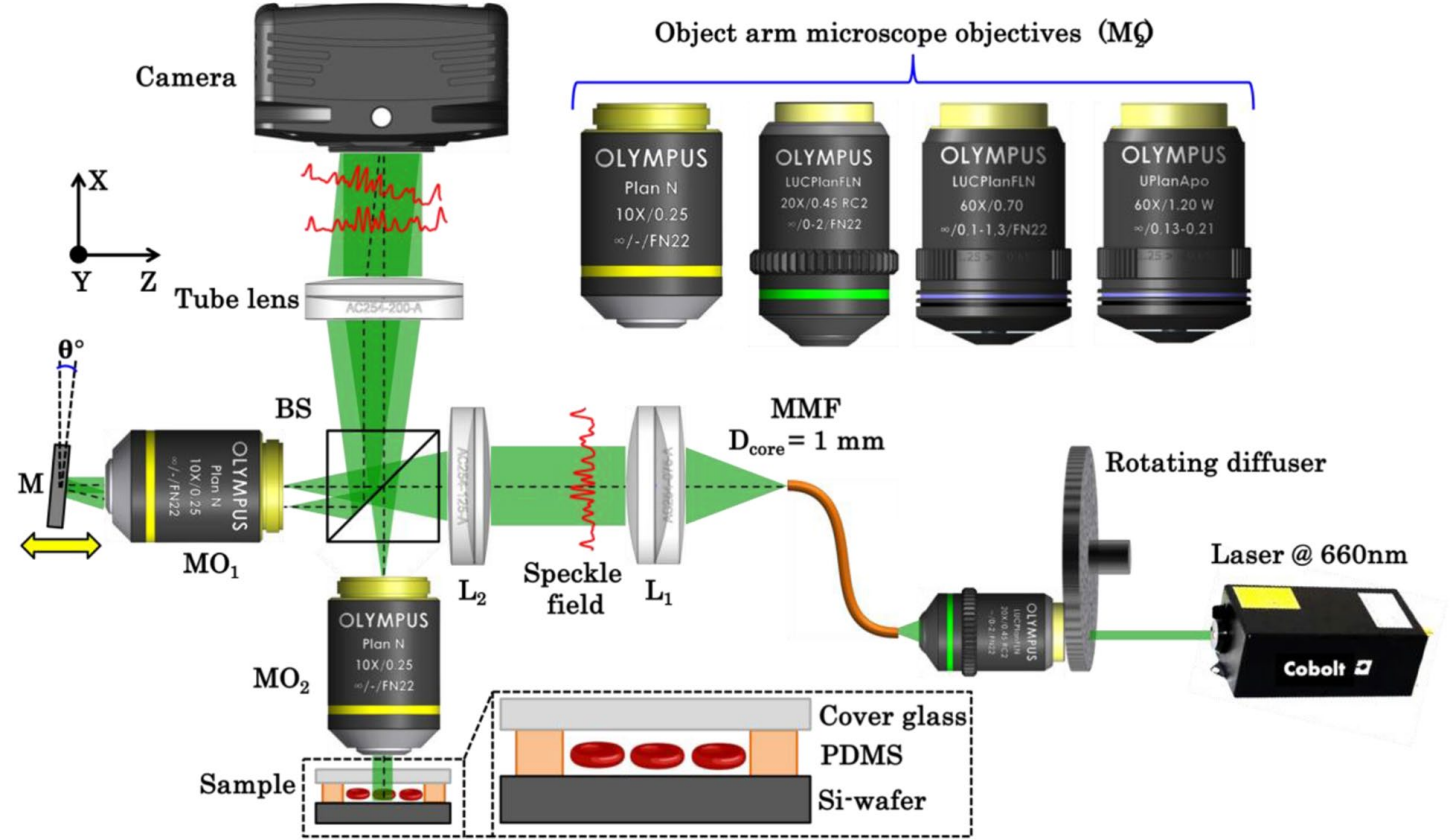
Schematic diagram of the DSI based IM system. *MO*_*1–2*_ microscope objectives, *BS* beam splitters, *L*_*1–2*_ lenses, *MMF* multi-mode fiber, *M* mirror and *CMOS* complementary metal oxide semiconductor camera.

The experimental scheme of laser speckle or DSI based IM system is illustrated in Fig. 8. The optical configuration is a Linnik interference microscopy system. The laser light beams coming from Cobolt Flamenco (λ = 660 nm; Δλ = 0.001 pm) illuminated the rotating diffuser (RD) with a beam (spot size at diffuser plane ~ 1 mm). The RD generated temporally varying statistically independent speckle fields, which added on the basis of intensity. The speed of the RD also plays an important role which controls the spatial coherence properties of the output field^25^. The scattered photons at the output of RD are directly coupled into a multi-mode fiber (Thorlabs: part # M35L01) using 20 ×/0.45NA objective lens as shown in Fig. 8. The core diameter of multi-mode fiber (MMF) is 1 mm. The RD followed by MMF generated uniform illumination, i.e., speckle free field, at the output port of MMF, which acts as an extended purely monochromatic light source named DSI. Before hitting the RD, the laser beam has high spatial and high temporal coherence properties. The RD and MMF significantly changed the spatial coherence properties of the light field with minimal effect on the temporal coherence properties. Thus, the output of MMF has low spatial and high temporal coherence properties.

The output of MMF is attached to the input port of the interference microscopy system. The speckle field at the output of MMF is nearly collimated using lens L_1_ (fl: 75 mm). The second lens L_2_ (fl: 125 mm) focused the light beam at the back focal plane of the microscope objective MO_2_ to achieve nearly uniform illumination at the sample plane. The beam splitter BS split the input speckle field into two; one is directed towards the sample (S) and the other one towards the reference mirror. The light beams reflected from sample and mirror are recombined and overlapped at the detector using BS and a tube lens (TL).

It is highlighted in the simulation work that to obtain interference pattern the speckle fields coming from the object and reference arm must be correlated and should not be shifted more than the speckle size. If any one of the situations is not satisfied, then the interference pattern will not be observed. Thus, slight misalignment in the optical setup can completely wash out the interference pattern at the detector even though the DSI has high temporal coherence properties. The alignment of object and reference speckle fields is done with the help the reference mirror. The reference mirror is attached to the three axis precision controls stage: one translation and two angular motions. The translation stage adjusted the separation between MO_1_ and reference mirror and tries to bring the mirror within the depth of field of MO_1_. The kinematic mount (Thorlabs: part # KMS/M) is used to control the angle of the reference mirror along two angular directions. The translation and angular motions in the reference mirror helped to obtain correlated object and reference speckle fields at the detector. The translation of the reference mirror controlled the correlation between the speckle fields and helped to obtain high contrast fringes. The angular motion of reference mirror controlled the angle between the object and reference field or the fringe density in the camera FOV. When the angular position of the mirror is changed, the decorrelation between the speckle fields took place due to the shift in the superimposed speckle fields.

As mentioned in the section “Superposition of the shifted speckle fields”, the interference fringes can be completely washout if the shift between the speckle fields is greater than or equal to the speckle size. The shift between the speckle fields is then adjusted using the translation stage to get maximum correlation or fringe contrast. Thus, three axis precision controlled the position and the orientation of the mirror and helped to obtain high contrast specklogram with variable fringe density. Due to the high temporal and low spatial coherence properties of DSI, good quality interferograms are generated with variable fringe density over the entire camera FOV provided the fields are correlated at the detector.

The reference mirror and MO_1_ is attached to a motorized stage (Thorlabs: part # MTS50-Z8) of the translation range of 50 mm. The combined motion of the mirror and MO_1_ unit adjusted the OPD between the object arm and reference arm. This way the curvature of the interference fringes is controlled while employing different objective lenses in the object arm as illustrated in Fig. 6.

## Supplementary Information

Supplementary Information.

## Data Availability

The authors declare the availability of the data used in the research to obtain the results reported in the manuscript upon reasonable request.
