# Supplementary material for: Demystifying speckle field interference microscopy
Source: Sci Rep. 2022 Jun 27;12:10869. doi: 10.1038/s41598-022-14739-0 (PMC9237063; doi:10.1038/s41598-022-14739-0)
Supplement: Supplementary file 1 — Supplementary Information. [file 41598_2022_14739_MOESM1_ESM.docx]

**Supplementary Materials for**

**Demystifying speckle field interference microscopy**

Azeem Ahmad^1,†^, Nikhil Jayakumar^1,†^, and Balpreet Singh Ahluwalia^1,2^

^1^Department of Physics and Technology, UiT The Arctic University of Norway, Tromsø 9037, Norway

^2^Department of Clinical Science, Intervention and Technology Karolinska Institute, and Center for Fetal Medicine, Karolinska University Hospital, Stockholm 17177, Sweden

*Corresponding author: ahmadazeem870@gmail.com, balpreet.singh.ahluwalia@uit.no

^†^These authors contributed equally to this work.

1. **Comparison of different light sources**

In the early days of interferometry, thermal light sources/white light (WL) spatially filtered with the help of pinhole are being employed due to absence of pure monochromatic light sources like lasers. The pinhole improves the spatial coherence of the light source at the expense of huge intensity loss. Thus, restricts the practical applications of optical interferometry techniques such as QPM and DHM. In addition, WL source has large spectral bandwidth, i.e., composition of large number of monochromatic spectral components, which confines the interference fringe in a limited interference field of view (iFOV) of the camera Figure S1(a). Moreover, it also restricts the implementation of IM only for a fixed objective lens as identical objective lenses are required in both object and reference arm to match the optical path length within the temporal coherence (TC) length (1 – 2 µm) of the light source. The iFOV can be increased by employing either a narrow bandpass light emitting diode or inserting a spectral filter in the WL beam path, also called filtered white light (FWL), at the cost of further intensity loss (see Figure S1(b)). On the contrary, narrowband lasers overcome the restriction of the limited iFOV as in case of WL/FWL (Figure S1(c)). However, it degrades the image quality due to the presence of coherent noise and parasitic fringes which are generated due to the large TC length of the lasers. As a consequence, it reduces the spatial phase sensitivity and height measurement accuracy of the system. Contrary to the conventional light sources, DSI has high TC length almost equal to the TC length of the parent laser light source and low spatial coherence (SC) length depending on the source size. High TC and low SC length of DSI helps to achieve coherent noise free interference pattern over the whole camera FOV unlike conventional light sources (Figure S1(d)).

**Figure S1. Comparison of conventional light sources.** a) white light using halogen lamp, b) light emitting diode or filtered white light, c) laser and d) DSI also called pseudo-thermal light source (PTLS). The comparison in terms of the extent of iFOV, quality of the interference fringes and spatial phase sensitivity. ∆λ is the spectral bandwidth and σ is representing the spatial phase noise or the spatial phase sensitivity of the interferometric system. The line profiles along the full FOV are depicted in red color corresponding to all types of light sources. TC and SC represent the temporal coherence and spatial coherence of the light source, respectively.

1. **Speckle theory**

The superposition of multiple randomly phased complex components results into an irregular pattern in 3D space called speckle pattern, which is a granular like structure [1]. Mathematically, the resultant phasor $A\left( x,y,z \right)$ at a single point in space–time can be represented as follows [1]:

$$A\left( x,y,z \right)=\frac{1}{\sqrt{N}}\sum_{k=1}^{N} a_{k}e^{i\phi_{k}}=Ae^{j\theta} (S1)$$

where, N is the total number of randomly phased complex components of amplitudes $\frac{a_{k}}{\sqrt{N}}$ and the phases $\phi_{k}$. A and θ are the amplitude and phase of the resultant phasor, respectively.

In general, if we have two or more speckle patterns then their sum can be done either on the basis of amplitude or intensity. The speckle pattern summed on the amplitude basis does not change the statistical distribution of the amplitude and intensity both [1]. Thus, the speckle contrast of the final speckle pattern does not reduce. On the contrary, addition on the intensity basis does reduce the fluctuation or contrast of the speckle provided some degree of decorrelation exist between the speckle patterns being added [1]. If the detector integrates M1 uncorrelated or statistically independent speckles, then the response of the detector is the summation of their intensity patterns. Thus, the total intensity is given by the sum

$$I_{T}=\sum_{m=1}^{M} I_{m}=\sum_{m=1}^{M} \left| A_{m} \right|^{2} (S2)$$

The correlation between the nth and mth intensity patterns is represented by the following expression [1]

$$C_{\mathrm{nm}}=\frac{\left\langle I_{n}I_{m} \right\rangle-\left\langle I_{n} \right\rangle\left\langle I_{m} \right\rangle}{\left[ \left\langle\left( I_{n}-\left\langle I_{n} \right\rangle\right)^{2} \right\rangle\left\langle\left( I_{m}-\left\langle I_{m} \right\rangle\right)^{2} \right\rangle\right]^{\frac{1}{2}}} (S3)$$

The value of correlation lies between 0 and 1. The correlation equal to 1 and 0 corresponds to the statistically dependent (unreduced speckle contrast) and independent (reduced speckle contrast) speckle pattern intensities, respectively. The contrast of the averaged image depends on the number of speckle patterns (M_1_) being added and defined by$k=\frac{1}{\sqrt{M_{1}}}$. Thus, sufficiently large number of uncorrelated speckle patterns must be averaged to observe the speckle free imaging. The correlation between the speckle pattern depends on several factors like amount of translation/rotation of rough surface, geometry of the rough surface and size of the speckle [1].

The speckle patterns can be classified into two categories called objective and subjective speckle. The objective speckles are obtained on the screen if a lens is not involved in the optical configuration. Whereas, the speckle patterns formed at the image plane of the optical system that incorporates a lens are subjective speckle.

The average size of the objective speckle on the observation plane is given by [1, 2]

$$d_{o}=\frac{\lambda z}{D} (S4)$$

where, $\lambda$ is the illumination wavelength, z is the distance between the rough surface and observation plane and D is the beam diameter at the rough surface. For subjective speckles, the average speckle size at the image plane is given by the following expression [2]

$$d_{s}=1.22\left( 1+M \right)\lambda F (S5)$$

where, M is the magnification of the optical system and F is the f-number (focal length/effective aperture) of a lens.

1. **Dynamic speckle interferometry**

In the paraxial wave approximation, the complex amplitude of the speckle field at a point $(x,y)$ in the observation plane is related to the complex amplitude $A(\xi,\eta)$ of the scattered wave field at a location $(\xi,\eta)$ right after the scattering surface plane by the following Fresnel diffraction integral [1]:

$$A\left( x,y \right)=\frac{e^{\mathrm{jkz}}}{j\lambda z}e^{j\frac{k}{2z}\left( x^{2}+y^{2} \right)}\iint_{-\infty}^{\infty} A(\xi,\eta)e^{j\frac{k}{2z}\left( \xi^{2}+\eta^{2} \right)}e^{-j\frac{2\pi}{2z}\left( x\xi+y\eta\right)}d\xi d\eta(S6)$$

The above Equation (S6) is simply the Fourier transform of the product of the complex amplitude right after the rough surface and a quadratic phase factor.

The complex amplitude $A(\xi,\eta)$ of the scattered wave field at the scattering surface

$$A\left( \xi,\eta\right)=Ae^{j\phi(\xi,\eta)} (S7)$$

where, $\phi(\xi,\eta)$ is the random phase introduced in the incident wave due to the scattering surface.

The superposition of two speckle fields, on-axis and tilted, at a particular instant of time, say $A_{1}\left( x,y \right)$ and $A_{2}\left( x,y \right)$, can be represented by the following relation:

$$H\left( x,y \right)=\left| A_{1}\left( x,y \right)+A_{2}\left( x,y \right)e^{j2\pi\left( f_{x}x+f_{y}y \right)} \right|^{2} (S8)$$

where, $H\left( x,y \right)$ is the intensity pattern generated due to the superposition of two speckle fields at the detector also called specklogram. $f_{x}$ and $f_{y}$ are the global spatial frequencies of the speckle field $A_{2}\left( x,y \right)$ along x and y axis and can be given as follows:

$$f_{x}=\frac{cos(\theta_{x})}{\lambda}; f_{y}=\frac{cos(\theta_{y})}{\lambda} (S9)$$

where, $\theta_{x}$ and $\theta_{y}$ are the angles of the propagation direction of the speckle field from x and y axis. The intensity patterns of the speckle fields $A_{1}\left( x,y \right)$ and $A_{2}\left( x,y \right)$ can be calculated by the following relations

$$I_{1}(x,y)=\left| A_{1}\left( x,y \right) \right|^{2}; I_{2}(x,y)=\left| A_{2}(x,y) \right|^{2} (S10)$$

The specklogram is completely filled of speckle noise which can be reduced by either rotating or translating the rough surface or diffuser. The rotating diffuser generates temporally varying speckle patterns. If large numbers of temporally varying speckle fields are averaged within the integration time ‘$T$’ of the detector, then the integrated intensity can be represented as follows:

$$I_{T}=\frac{1}{T}\int_{0}^{T} H(t)dt (S11)$$

The average (Equation S11) of large number of unstable and stable specklograms corresponding to different speckle fields, form a constant background and nicely oriented interferogram, respectively. Mathematically, it can be written as follows:

$$I_{T}=\left\{ \begin{matrix} const or mean of I_{T} & C_{12}=0 \\ \mathrm{Interferogram} & C_{12}=1 \end{matrix} \right\} (S12)$$

where, $C_{12}$ is the correlation between the speckle fields $A_{1}\left( x,y \right)$ and $A_{2}\left( x,y \right)$ being superimposed to generate specklograms. If the speckle fields are identical, i.e., exact replica, then they exhibit the highest correlation and considered correlated to each other. On the other hand, uncorrelated speckles correspond to the non-identical speckle fields. In other words, Equation S12 implies that if the speckle fields are uncorrelated ($C_{12}=0$) then interference pattern will not be observed. To generate high contrast interferograms, the speckle fields must be correlated ($C_{12}=1$) to each other. The correlation values in between 0 and 1 corresponds to the reduced fringe visibility of the resultant interferogram.

Furthermore, two identical fields can also exhibit decorrelation when one of the speckle field is shifted or translated (say by ‘$\Delta x;\Delta y$’) with respect to the other one. The extent of the shift within which the fields are still correlated is called correlation length ‘$l_{c}$’ and is decided by the speckle size (Equations S4 and S5). Thus, the two identical speckle fields do not form the interference pattern if the translation is greater than the correlation length/speckle size in one of the speckle fields. For identical speckle fields, Equation (S12) in terms of shift or transition can be modified as follows:

$$I_{T}=\left\{ \begin{matrix} const or mean of I_{T} & C_{12}=0 or \Delta x;\Delta y\geq l_{c} \\ \mathrm{Interferogram} & \begin{matrix} C_{12}=1 or \Delta x;\Delta y=0 \\ 0<C_{12}<1 or \Delta x;\Delta y<l_{c} \end{matrix} \end{matrix} \right\} (S13)$$

1. **Simulation of diffuser and speckle field**

In the simulation, a rough object of size of 512 × 512 pixels having height variations h(x,y) is created as shown in Figure S2.

**Figure S2.** (a) and (b) 2D and 3D views of the simulated rough surface being used to generate speckle fields for the simulation studies. The phase values of the rough surface vary from – pi to + pi.

The height variation is chosen in a way such that it corresponds to the phase variation uniformly distributed between – π to + π. The phase variation of the rough surface is related to its height variation by the following relation:

$$\phi\left( x,y \right)= \frac{2\pi}{\lambda}h\left( x,y \right) (S14)$$

The transmittance of the diffuser is given by the following expression:

$$T\left( x,y \right)=T_{0}\left( x,y \right)\exp\left( i\frac{2\pi}{\lambda}h\left( x,y \right) \right) (S15)$$

where, $T_{0}\left( x,y \right)$ is the transmission coefficient of the diffuser, $\lambda$ is the wavelength of the incident light.

Let us consider a circular light beam of unity amplitude and diameter D hits the diffused surface. Numerically, it can be done by multiplying the transmittance given in Equation S15 with a circular mask (diameter: D) having 1s inside the circle and 0s outside the circle as illustrated in Figure S3. The speckle pattern is then generated by performing its Fourier transform (Equation S6) and multiplying element wise by the complex conjugate [3].

**Figure S3.** (a) Simulated rough surface being used to generate speckle fields for the simulation studies. (b) Binary mask with a circular opening of diameter ‘D’. The diameter of the opening decides the laser beam diameter hitting the rough surface. (c) Multiplication of the rough surface and binary mask to limit the contribution of the scattering sites falling within the opening region of the mask on the resultant speckle pattern. This way it mimics the experimental situation where the scattering sites interacting with the laser only contribute to the resultant speckle field.

1. **Superposition of correlated and uncorrelated speckle fields as a function of global phase fronts**

In optical interferometry, the speckle patterns are called correlated or statistically dependent if they match elementwise (or pixel-wise) with each other. The uncorrelated speckle patterns do not match element-wise with each other and also called statistically independent. The correlation between the speckle patterns is calculated by employing Equation S3. Generation of uncorrelated speckle patterns is needed to reduce the speckle noise from the images. On the contrary, in the field of interferometry the speckle patterns must be correlated or statistically dependent to generate interference pattern due to their superposition at the detector.

To understand the effect of correlated and uncorrelated speckle fields with different global phase fronts on the resultant speckle pattern, the optical configurations depicted in Figure S4(a) are considered. It can be seen from Figure S4(a, b) that the global phase fronts (black dotted lines) of the two speckle fields (say R and O) have spherical shape before the tube lens (TL). The TL collimates both the speckle fields and overlaps them at plane P. In Figure S4(a), both the beams have same curvature (say C_­­1_) and different speckle fields (S_1_ and S_2_) before TL. It is observed that the resultant intensity pattern does not have any fringe like pattern as illustrated in Figure S4(a). Thus, statistically independent fields do not form nicely oriented interference pattern. On the contrary, the superposition of two correlated speckle fields form a nicely modulated intensity pattern at plane P as depicted in Figure S4(b). Note that the speckle noise does not affect the global shape of the modulated intensity pattern.


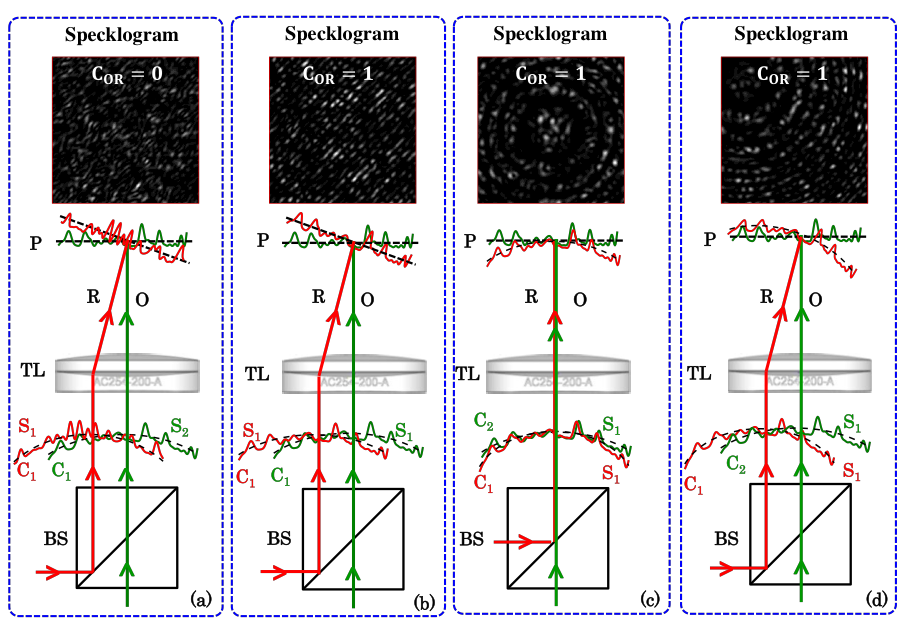


**Figure S4. Optical configurations to superimpose the correlated and uncorrelated speckle fields.** (a, b) represent the superposition of two uncorrelated and correlated speckle fields in off-axis configuration, respectively. The global phase fronts (black dotted lines) are assumed to be plane wavefronts; on-axis and tilted as shown in Figure S4(a, b). (c) and (d) represent the superposition of two correlated speckle fields in the on-axis and off-axis configuration, respectively. The global phase fronts of the speckle fields are spherical and plane. The shape of the global phase fronts (black dotted lines) does not the affect the quality/contrast of the specklograms. It only changes the shape of the fringes in the resultant pattern.

Next, we investigated the case when two correlated speckle fields (S1) with different global spherical phase fronts (C_1_ and C_2_ before TL) are superimposed at the focal plane of TL as shown in Figure S4(c). This is an on-axis superposition of two speckle fields. TL collimates one of the speckle fields having curvature C_2_, whereas second speckle field has slight curvature after TL. This generates specklogram with spherical fringes centered at the origin as shown in Figure S4(c). Figure S4(d) illustrates the off-axis superposition of two correlated speckle fields with global phase fronts C_1_ and C_2_. The beam R meets the beam O at an angle as depicted in Figure S4(d). The superposition of these two speckle fields generates off-centered circular fringes in the specklogram as shown in Figure S4(d). This concludes that the shape of the global phase fronts does not reduce the quality of the specklogram and fringe shape as long as the speckle patterns are correlated.

**Figure S5.** The average images of 360 specklograms for a speckle size of 15 pixels corresponding to different shifts in one of the speckle fields being superimposed with another identical unshifted speckle field. The shift is done sequentially from 0 pixel to the average speckle size in a step of 1 pixel.

**Figure S6.** The average images of 360 specklograms for a speckle size of 30 pixels corresponding to different shifts in one of the speckle fields being superimposed with another identical unshifted speckle field. The shift is done sequentially from 0 pixel to the average speckle size in a step of 2 pixel.

**Figure S7.** (a) Average interferogram of the 360 simulated statistically independent specklograms corresponding to different rotation angles of the diffuser from 1° to 360° in a step of 1°. The specklograms are generated due to the supposition of two correlated speckle fields (see main text Figure 3 for more details) of speckle size of 30 pixels. (b) The line profile of the average image called interferogram along the green dotted horizontal line. It can be seen that modulation depth of the interferogram is not constant over the entire FOV. This could be due to the insufficient averaging of the statistically independent specklograms. This can be improved by generating large number of statistically independent specklograms.

**Table S1. The normalized correlation between the object and reference arm speckle fields as a function of OPD between them.**

| **OPD (mm)** | **C_OR_** | **OPD (mm)** | **C_OR_** | **OPD (mm)** | **C_OR_** | **OPD (mm)** | **C_OR_** | **OPD (mm)** | **C_OR_** |
| --- | --- | --- | --- | --- | --- | --- | --- | --- | --- |
| 0 | 0.70 | 6 | 0.73 | 12 | 0.74 | 18 | 0.76 | 24 | 0.75 |
| 1 | 0.72 | 7 | 0.75 | 13 | 0.75 | 19 | 0.73 | 25 | 0.75 |
| 2 | 0.72 | 8 | 0.73 | 14 | 0.75 | 20 | 0.76 | 26 | 0.75 |
| 3 | 0.73 | 9 | 0.73 | 15 | 0.74 | 21 | 0.74 | 27 | 0.73 |
| 4 | 0.75 | 10 | 0.71 | 16 | 0.73 | 22 | 0.74 | 28 | 0.76 |
| 5 | 0.74 | 11 | 0.73 | 17 | 0.74 | 23 | 0.76 | 29 | 0.71 |

**Figure S8.** (a) High fringe density interferometric image of USAF resolution chart while using 10×/0.25NA in the reference arm and 60×/0.7NA objective lens in the object arm. The insets depict the zoomed view of the region marked with red dotted box. (b) Single-shot recovered phase image of the resolution chart corresponding to aforementioned objective lenses. The color bar is in rad.

1. **References:**

1. J. W. Goodman, *Speckle phenomena in optics: theory and applications* (Roberts and Company Publishers, 2007).

2. J. C. Dainty, *Laser speckle and related phenomena* (Springer science & business Media, 2013), Vol. 9.

3. J. W. Goodman, *Introduction to Fourier optics* (Roberts and Company Publishers, 2005).
